# Allergic contact dermatitis to clostridiopeptidase A with a nummular eczema‐like spread

**DOI:** 10.1111/cod.13982

**Published:** 2021-10-18

**Authors:** Caterina Foti, Gianluca Calianno, Silvia Mazzotta, Fabrizio Guarneri, Paolo Romita

**Affiliations:** ^1^ Department of Biomedical Science and Human Oncology, Dermatological Clinic University of Bari Bari Italy; ^2^ Department of Clinical and Experimental Medicine University of Messina Messina Italy

**Keywords:** allergic contact dermatitis, case report, chloramphenicol, clostridiopeptidase A, collagenase I, leg ulcers, nummular eczema

Topical medications used for the treatment of venous leg ulcers are a well‐known cause of allergic contact dermatitis (ACD).^1^ Among these, topical drugs containing proteolytic enzymes (used for wound debridement) are rarely involved.^2^, ^3^ We herein report a case of severe ACD caused by clostridiopeptidase A, also known as collagenase I.

## CASE REPORT

**FIGURE 1:**
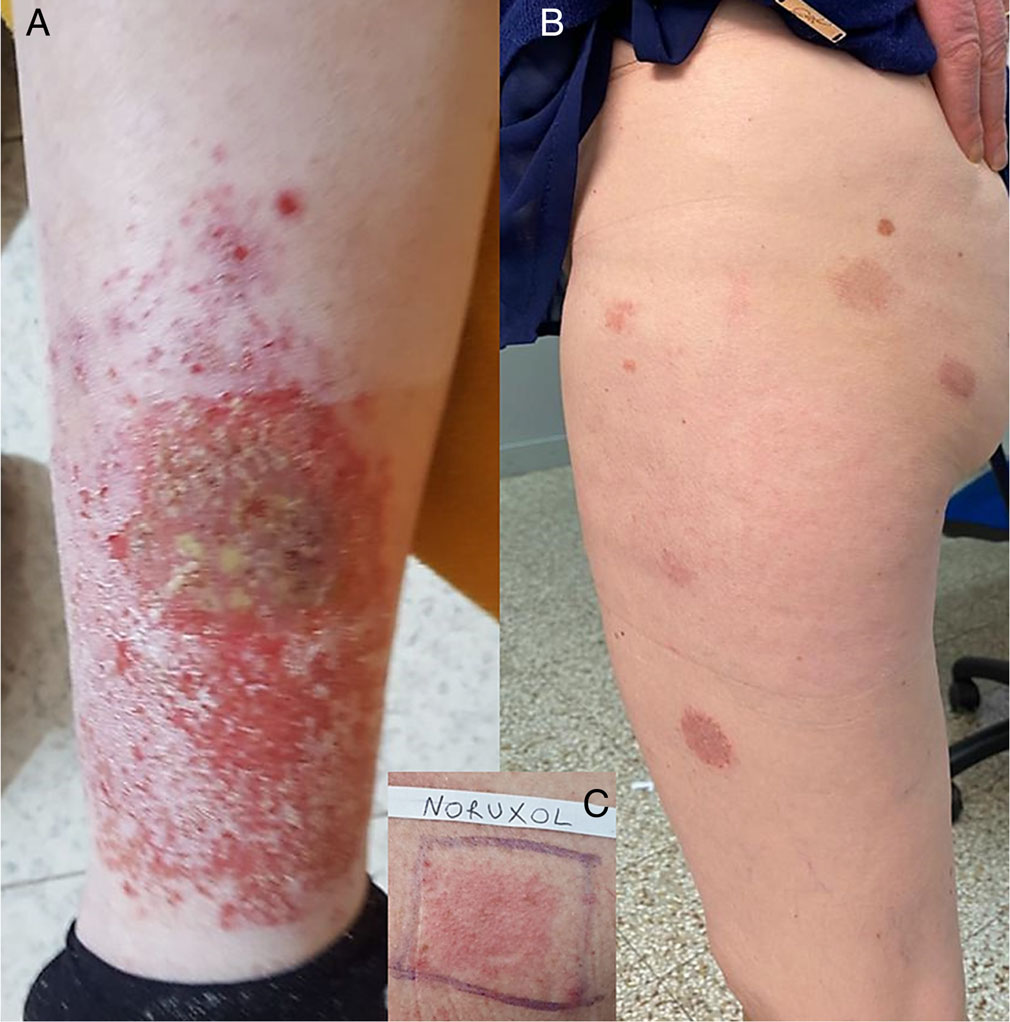
(**A)** Eczematous lesions around the leg ulcer and (**B**) nummular eczema‐like spreading; (**C**) positive patch test to Noruxol “as is” on day 4

A nonatopic 64‐year‐old woman presented to our attention for the onset of a diffuse crusted eczematous dermatitis with nummular aspects (Figure 1). The patient reported that the eruption had started to appear about 2 weeks after the application of a topical ointment containing chloramphenicol 1% and clostridiopeptidase A 60 U.I. (Iruxol, Smith & Nephew GmbH, Hamburg, Germany) under occlusion with a zinc oxide bandage, prescribed by her physician to treat a venous ulcer on her left leg. The skin lesions appeared around the leg ulcer and then spread within 3 weeks to both lower limbs, the upper limbs, and trunk, with nummular eczema‐like aspects. Her physician already prescribed oral therapy with amoxicillin–clavulanate (875 + 125 mg twice daily) for 6 days; however, there was no improvement of the skin lesions. Use of the topical ointment was immediately discontinued, and topical application of mometasone furoate once daily resulted in healing of the eczematous lesions in 2 weeks. Four weeks after the resolution of the dermatitis, patch tests were performed with the SIDAPA (Società Italiana di Dermatologia Allergologica Professionale e Ambientale) baseline series (FIRMADiagent, Florence, Italy), a piece of the zinc oxide bandage, and the ointment used by the patient. Patch tests were applied on the back and left under occlusion for 2 days with Haye's Test Chambers (Haye's Service B.V., Alphen aan den Rijn, The Netherlands) on Soffix tape (Artsana, Grandate, Italy). Readings were performed on day (D) 2 and D4 according to guidelines,^4^ and showed a positive reaction to paraphenylenediamine (++, past relevance) and Iruxol ointment (++). Subsequently, patch tests were performed with chloramphenicol 5% pet. and Noruxol (containing clostridiopeptidase A, liquid paraffin, and white petrolatum; Smith & Nephew GmbH, Hamburg, Germany), liquid paraffin, and white petrolatum, yielding positivity only to Noruxol (++; Figure 1). Based on these findings, we diagnosed ACD to clostridiopeptidase A. Ten healthy individuals were patch tested with Iruxol and Noruxol with negative results.

## DISCUSSION

ACD caused by topical medications is a well‐known, but probably underestimated and misdiagnosed complication.^5^ We herein reported a case of severe ACD caused by clostridiopeptidase A, a proteolytic enzyme widely used in wound and ulcer care. While chloramphenicol is a well‐known sensitizer,^6^ clostridiopeptidase A has rarely been reported as the culprit of ACD.^2^, ^3^ Nummular eczema is usually a manifestation of atopic dermatitis, but contact allergy has also been demonstrated to be associated with nummular eczema‐like aspects.^7^ To our knowledge, this is the first case of ACD to clostridiopeptidase A with a nummular eczema‐like spread.

We speculate that the application of the medication under occlusion could have favoured contact sensitization and lymphatic and/or hematogenous dissemination, which are proposed mechanisms for ACD spreading.^8^

Beyond the peculiar clinical manifestations, our case highlights that contact sensitization to topical medications containing clostridiopeptidase A, although rare, should be always borne in mind by physicians.

## CONFLICTS OF INTEREST

All the authors have no conflict of interest to declare.

## AUTHOR CONTRIBUTIONS

**Caterina Foti:** Conceptualization (equal); data curation (equal); supervision (equal); writing – review and editing (equal). **Gianluca Calianno:** Conceptualization (equal); data curation (equal). **Silvia Mazzotta:** Data curation (equal); formal analysis (equal). **Fabrizio Guarneri:** Data curation (equal); formal analysis (equal); supervision (equal). **Paolo Romita:** Conceptualization (equal); data curation (equal); formal analysis (equal); supervision (equal); validation (equal); writing – review and editing (equal).
